# Designing a Recombinant Multi-Epitope Antigen of *Echinococcus granulosus* to Diagnose Human Cystic Echinococcosis

**Published:** 2020

**Authors:** Aliyar MIRZAPOUR, Seyyed Javad SEYYED TABAEI, Mojgan BANDEHPOUR, Ali HAGHIGHI, Bahram KAZEMI

**Affiliations:** 1. Department of Medical Parasitology and Mycology, International Branch of Shahid Beheshti University of Medical Sciences, Tehran, Iran; 2. Department of Parasitology and Mycology, School of Medicine, Mashhad Branch, Islamic Azad University, Mashhad, Iran; 3. Department of Medical Parasitology and Mycology, School of Medicine, Shahid Beheshti University of Medical Sciences, Tehran, Iran; 4. Cellular and Molecular Biology Research Center, Shahid Beheshti University of Medical Sciences, Tehran, Iran; 5. Department of Biotechnology, School of Advanced Technologies in Medicine, Shahid Beheshti University of Medical Sciences, Tehran, Iran

**Keywords:** Multi-epitope, *Echinococcus granulosus*, Hydatidosis, ELISA

## Abstract

**Background:**

Cystic echinococcosis can cause severe disease and probable death in humans. Epitopes of its antigens play a key role in the sensitivity and specificity of immunodiagnostic tests.

**Methods:**

Epitope prediction software programs predict the most antigenic linear B-cell epitopes of AgB (8 kD), Ag5, and Ag95. Six such epitopes were predicted and connected by “Gly-Ser” linker and synthesized. The purity of the concentrated recombinant multi-epitope protein was assessed by 15% SDS-PAGE. Overall, 186 serum samples were collected from the Loghman Hakim Hospital and different laboratories, Tehran, Iran, from July 2016 to February 2017. Patients infected with hepatic hydatid cysts, patients infected by other parasites and viruses, and healthy individuals were used to detect the anti-CE IgG using recombinant multi-epitope protein.

**Results:**

Forty-one samples out of 43 cases of hydatidosis were diagnosed correctly as positive, and two were negative. In addition, six negative cases of healthy individual group were diagnosed as positive and negative with rMEP-ELISA and the commercial kit, respectively. Therefore, these six samples were considered as false positive using our method. In addition, a diagnostic sensitivity of 95.3% (95% CI, 84.19% to 99.43%) and a specificity of 95.0% (95% CI, 89.43% to 98.14%) were obtained using optimum cutoff value (0.20). The sensitivity and specificity of the commercial kit was 100%.

**Conclusion:**

Our findings showed high diagnostic accuracy of the ELISA test using the developed recombinant protein, which encourages the use of this recombinant multi-epitope protein for rapid serological diagnosis of hydatidosis.

## Introduction

Cystic echinococcosis (CE) is a zoonotic disease caused by a parasite of the class Cestodes, genus *Echinococcus.* Canids are the only host for the adult worm of *E. granulosus*. Adult tapeworms attach to the intestinal epithelium and sexually reproduce towards the development of eggs (1). Natural intermediate hosts, such as humans, cattle, and sheep, contract the infection by ingesting the eggs. Liver is the major site of cystic hydatid (50%–70% of all cysts), but it can also develop in lungs (20%–30%) and sometimes in spleen, kidneys, bone, brain, and rarely in other organs (2, 3).

CE can cause severe disease and probable death in humans as well as financial burden of late diagnosis, treatment costs, and livestock-associated losses (4, 5). CE is endemic in Asia, Africa, Eastern Europe, South and Central America, the Middle East, Australia, and New Zealand (6). In endemic areas, the incidence rate of Human CE has been recorded at >50 per 100,000 persons/year, and a 10%–50% prevalence rate in parts of Peru, Argentina, East Africa, central Asia, and China (7, 8). The clinical manifestations of CE are not critically pathognomonic; hence, searching for an accurate diagnostic method is vitally important (3, 9).

Ultrasonography (US), computerized tomography (CT), and magnetic resonance imaging (MRI) are the main primary diagnostic methods for CE (3, 10). US is used for the diagnosis of both hepatic and extrahepatic CE (11), while CT is used to detect the presence of daughter cysts and calcification in the cyst wall (12). MRI reveals the structures of the CE (8). These diagnostic tests are not precise enough and must be confirmed by a specific technique. Serological tests, based on the detection of antibodies in the patient’s serum, are sensitive in the diagnosis of most of the pathogenic infections (13). Nowadays, some sensitive serodiagnostic assays such as ELISA, immunoblot (IB), and indirect immunofluorescent antibody test (IFA) have replaced some old serological methods (14). For the detection of infectious diseases, e.g. CE, ELISA is recommended as a high-sensitivity assay (15).

The standardization of antigenic preparations is the major complication in serodiagnosis of CE. Hence, it is necessary to use an appropriate source of antigenic material derived from different growing phases of *E*. *granulosus* (16). According to previous researches, AgB (8 kDa), Ag5, and Ag95 lipoproteins are the most important antigens for serodiagnosis of CE (17–20).

The sensitivity and specificity of immunodiagnostic tests depend on the antigenicity and conservation of epitopes of antigens, respectively. The prediction of immunogenic epitopes on proteins’ surface is essential to design an immunodiagnostic test. Continuous linear epitopes predicted by epitope prediction procedures utilize protein sequences as input data. Amino acid properties, comprising immunoinformatic analysis and prediction of B-cell epitopes, form the basis of prediction methods (21, 22). Bioinformatics approaches comprise a new technique to search for microorganism vaccines and antigens used to diagnose infections.

In this research, we predicted B-cell epitopes of AgB (8 kDa), Ag5, and Ag95 using bioinformatics approaches and produced a recombinant protein, used for serological diagnosis of *E. granulosus.* The recombinant protein was purified, and its diagnostic efficacy was assessed using ELISA and immunoblotting.

## Materials and Methods

### Computer modeling prediction of immunodominant epitopes and construction of rMEP expression plasmid

The sequences of amino acids of the AgB (8 kDa), Ag5, and Ag95 of *E. granulosus* were retrieved from the National Center for Biotechnology Information (NCBI) Database. IEDB, Bepipred (http://www.cbs.dtu.dk/services/BepiPred/), and ABCpred (http://crdd.osdd.net/raghava/abcpred/) are the epitope prediction software programs, used to predict the most antigenic linear B-cell epitopes of the fusion protein. Finally, BLASTP were used to prove the presence or absence of predicted epitopes. Briefly, six predicted B-cell epitopes of the antigens were connected using a “Gly-Ser” linker resulting in recombinant multi-epitope peptide (rMEP), and a His-tag was added at the end of the sequence. The sequence was synthesized by Gene Ray Biotech (Shanghai, China). It was then cloned into the bacterial expression vector pET-26b to produce recombinant expression plasmid pET-MEP.

### Expression of recombinant multi-epitope polypeptide

**Fig. 1:**
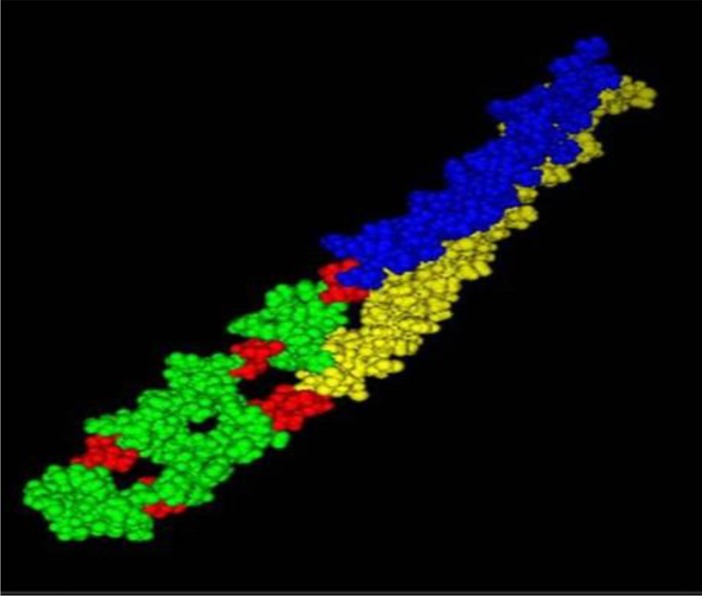
Predicted tertiary structure of the rMEP. This image was created using pyMOL software

*E. coli* strain BL21 (DE3) was transformed with the pET-MEP and cultured in Luria Bertani broth containing 100μg/ml ampicillin. The transformant was cultured overnight at 37 °C in a shaker incubator at 160 rpm. Afterward, it was subcultured into LB medium and incubated at 37 °C in a shaker incubator at 200 rpm. The logarithmic-phase culture (at OD_600_=0.6) was induced by 1 mM isopropyl-β-D-thiogalactopyranoside (IPTG) for 2, 6, and 12 h. Afterwards, un-induced and induced bacteria (of each time point) were used to analyze rMEP expression using SDS-PAGE. The gel was stained with Coomassie brilliant blue R-250. The secondary structure of the proteins was assessed using the SOPMA online software (https://npsa-prabi.ibcp.fr/cgi-bin/npsa_automat.pl?page=/NPSA/npsa_sopma.html). The B-cell epitopes of the three proteins were selected based on Emini surface accessibility, Kolaskar, Tongaonkar antigenicity, and Parker hydrophilicity (IEDB, BCEPRED, and ABCpred). Epitopes identified from AgB (8 kD), Ag5, and Ag95 (named Eg AgB_ EP1, Eg Ag5_EP1, Eg Ag5_ EP2, EgAg5_EP3, Eg Ag5_ EP4, and Eg Ag95_EP1 respectively) were used to construct a multiepitope peptide (Table 1). The tertiary structure of the rMEP was predicted by pyMOL software (Fig. 1).

**Table 1:** predicted B-cell Epitopes of Eg AgB (8 kD), Eg Ag5, and Eg Ag95 using three different bioinformatics applications

***Gene***	***Predicted epitope***	***Sequence***	***Position***
*Eg AgB* (8 kDa)	Eg AgB – EP1	LIMRKLGEIRDFFRSD	30 – 89
		PLGQKLAALGRDLTA	
		ICQKLQLKVHEVLKK	
		YVKDLLEEEDDDSK	
*Eg Ag5*	Eg Ag5 - EP1	DAEAEERGEYESER	169 – 182
	Eg Ag5 - EP2	EGTRTRNEQESDH	283 – 295
	Eg Ag5 - EP3	EKSRPISKPRRRRPT	359 – 373
	Eg Ag5 - EP4	WERRPQRPTS	386 – 395
*Eg Ag95*	Eg Ag95 - EP1	KGRGIETKTTESPLR	20 – 63
		KHFSLTLVGSQGIRL	
		SWEVQHLPSLQGTN	

### Polypeptide purification by affinity chromatography

His-tag was added at the end of the recombinant. A single colony was preinoculated in LB medium and sub-cultured. Afterward, the culture (OD _600_ = 0.6) was induced using IPTG (1mM) for 6 h at 37 °C. The cells were centrifuged at 12,000 rpm for 10 min at 5 °C. The pellet was then suspended and lysed in a lysis buffer (50 mM NaH2PO4-300mM NaCl, pH=8) containing 1 mM PMSF protease (Roche Applied Science) (23). The cell suspension was lysed by sonication on ice (power 400 W, 20 min, ultrasonic 3 sec, pause 5 sec for a loop), and the lysate was centrifuged at 7000 g for 30 min at 4 °C. The supernatant was then collected for purification. The supernatant was slightly shaken with 1 ml of Ninitrilotriacetic acid (NTA) resin (Novagen, USA). After the flow-through was collected, the column was washed three times with 10 ml wash buffer (50 mM NaH2PO4-300mM NaCl, pH 8), and the recombinant protein was eluted in 1 ml elution buffer (50 mM Tris, 0.5 M NaCl, and 250 mM imidazole). Bradford method was used to measure the concentration of purified recombinant protein, and the purity of the concentrated rMEP was assessed by 15% SDSPAGE.

### Confirmation of polypeptide by Immunoblotting

Electrophoresed protein was transferred on nitrocellulose membrane (Whatman, USA). The membrane was blocked with 5% skimmed milk in phosphate buffered saline (PBS) (pH 7.4) containing 0.1% Tween 20 (PBS-T) overnight at 4 °C. The membrane was washed several times with TBST (Tris-Buffered Saline-Tween 20), treated with Histag monoclonal antibody (diluted 1:10000 in 0.05% Tween-PBS) (Conjugated) (Dako Cytomation, Glostryp, Denmark), for one hour at room temperature, and then washed repeatedly.

The membrane was incubated with phosphatase substrate solution containing 0.3 gr/L nitroblue tetrazolium and 0.15 gr/L 5-bromo-4-chloro-3-indolylphosphate (NBT/BCIP) (PerkinElmer Life Sciences, Gaithersburg, MD, USA). Finally, the membrane was washed four times using distilled water to stop the reaction.

### Samples

Overall, 186 serum samples from three different clinical groups were collected; 1) 43 serum samples were collected from patients infected with hepatic hydatid cysts. The samples were confirmed using pathological methods after surgery; 2) 23 serum samples of patients infected by other parasites and viruses, including *Toxocara* (three samples), *Toxoplasma* (10 samples), *Taenia* (one sample), *Leishmania* (three samples), *Trichinella* (one sample), Malaria (one sample), HBsAg (two samples), HCV (two samples); and 3) 120 serum samples of healthy individuals were collected from the Loghman Hakim Hospital and different laboratories, Tehran, Iran, from July 2016 to February 2017. Non-CE patients and healthy donors were confirmed by total IgG commercial ELISA kit (Medizinische labor diagnostic AG, Germany Lot: E170424BW). The anti-CE antibody of all samples was assessed separately by both rMEP-ELISA (ELISA assay using our recombinant antigen) and the commercial ELISA kit, and the specificity and sensitivity of both assays were compared.

Detailed, informed consents were taken from all patients before participation in the study. The current experiment meets complete standards of the ethical conduct of research complied with the World Medical Association Declaration of Helsinki regarding ethical conduct of research involving human subjects and was approved by the Ethics Committee of Shahid Beheshti University of Medical Sciences.

For ELISA, several numbers of plates were pre-coated with 1 μg/well of the rMEP protein in carbonate buffer. Wells were washed five times using PBS supplemented with 0.05% Tween (PBS–T). The wells were blocked overnight. Using 200μL of blocking solution (BSA1%, 5% skimmed milk) at 4 °C. The wells were washed again, serum samples were added at 1:100 dilutions in dilution buffer (PBS-T, 0.25% BSA, 5% skim milk), incubated at 37 °C for 1 h, and then washed as mentioned. HRP-conjugated anti-human IgG antibody (Dako Cytomation, Glostryp, Denmark) (diluted 1:10000, stock concentration 13 g/L) was added and incubated at 37 °C for 1 h. Then, the wells were washed, and, 100 μl of substrate solution (3, 3′, 5, 5′-tetramethylbenzidine (TMB) and H _2_ O _2 _) was added to each well, and the plate was incubated for 15 min at room temperature. Totally, 50 μl of 2 N H2SO4 was added as stopping solution, and optical density (ODs) was read at 450 nm using ELISA reader (Anthos 2020, USA). All tests were performed in duplicate. The sera infected with other microorganisms were used to check cross-reactions.

### Statistical analysis

The cut-off value for positive reactions was calculated by mean ±2 SD of the absorbance of the 120 healthy individuals’ samples. The sensitivity was plotted against the level of one minus the specificity at each cutoff point on Receiver-Operating Characteristic (ROC) curve. Positive and negative serum samples were used to build the ROC curve. The zone under the ROC curve (AUC) is a standard amount of the accuracy of a diagnostic test. (SPSS ver. 19.0, Chicago, IL, USA) was used for statistical analysis. *P*-value<0.05 was considered as statistically significant.

## Results

### Identification and characterization of immunodominant epitopes

GenBank (https://www.ncbi.nlm.nih.gov/genbank/) was used to determine the nucleotide sequences of Ag5 (JF970202), antigen B (DQ835667), and EG95 (JF829212). Primary protein structure and the putative protein sequence of Ag B subunit, Ag 5, and Eg 95 were analyzed by Expasy tools (http://web.expasy.org/protparam/).

**Fig. 2:**
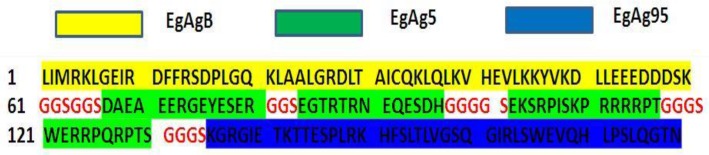
Amino acid sequence of the recombinant polypeptide in FASTA format Red sequences indicate“Gly-Ser” linker

The “Gly-Ser” linker was used to join the adjacent epitopes (Fig. 2).

**Fig. 3:**
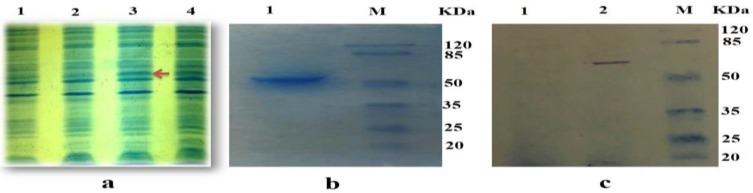
Preparation and identification of recombinant protein. **a**) SDS-PAGE of rMEP-induced expression with 1.0 mM IPTG for different times: lane 1, BL21 (negative control); lane 2, supernatant of IPTG-induced cells for 2 h; lane3, supernatant of IPTG-induced cells for 6 h; lane 4, supernatant of IPTG-induced cells for 12 h. **b)** The protein purification.**c,** SDS-PAGE of rMEP purification (M, marker; Lane 1, rMEP). **c)** Western blotting analysis: (M, marker; Lane 1, BL21 (negative control); Lane 2, purified recombinant protein)

The artificial multi-epitope gene was successfully synthesized by Gene Ray Biotech (Shanghai, China); PCR technique confirmed the construction of these plasmids, then transformed into *E. coli* BL21 (DE3) cells and expressed. Afterward, the induced cells were sonicated and analyzed by SDS-PAGE and stained with Coomassie brilliant blue. An expected band demonstrating the recombinant product was achieved (Fig. 3a). Ni–NTA affinity purification was used, and the purified products were analyzed by SDS-PAGE (Fig. 3b).

Moreover, western blot analysis confirmed the successful expression of the approximately 61 kDa protein, which was probably a dimer of the mentioned protein (Fig. 3c).

### ELISA using rMEP

**Fig. 4:**
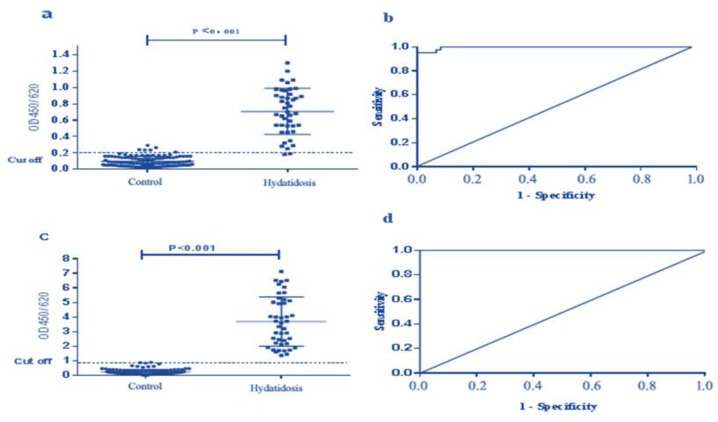
ELISA analysis of serum samples. The analysis was performed after considering positive control serum samples with surgical positive hydatidosis (43 sera) and negative control sera from patients with other diseases and healthy individuals (120 sera). **a)** Dot plot of the rMEP IELISA assay. **b)** ROC analysis of rMEP IELISA assay results. **c)** Dot plot of the Commercial kit ELISA assay results. **d)** ROC analysis of Commercial kit ELISA assay results

Indirect ELISA assay was performed on 186 serum samples by both rMEP-ELISA and Euroimmun commercial kit. A dot plot diagram outlined the OD values of these samples (Fig. 4a). Based on the ROC analysis, AUC for this test was 0.9964 (95% confidence interval (CI), 0.9914 to 1.001). In addition, a diagnostic sensitivity of 95.3% (95% CI, 84.19% to 99.43%) and a specificity of 95.0% (95% CI, 89.43% to 98.14%) were obtained from an optimum cutoff value (0.20). According to this cutoff value, 41 samples of 43 hydatidosis cases were diagnosed correctly as positive and two were negative. In addition, six negative cases of healthy individual group were diagnosed as positive and negative with rMEP-ELISA and the commercial kit, respectively. Therefore, these six samples were considered as false positive using our method. Additionally, all the healthy control samples were correctly diagnosed. Likewise, the commercial kit was utilized and compared with the fusion protein. The dot plot diagram is referred to in Fig. 4c, and the ROC curve is in Fig. 4d. AUC for the commercial kit was 1.000 (95% CI, 1.000 to 1.000), the sensitivity and specificity were 100% (95% CI, 91.78% to 100%) and 100% (95% CI, 96.97% to 100%), respectively, obtained from an optimum cutoff value (0.8). However, with the cutoff value, 43 of 43 hydatidosis cases were correctly diagnosed, including three healthy individuals diagnosed as borderline. We also have a cross-table with absolute numbers of positive and negative samples with these cutoff values. The positive/negative predictive values are also indicated (Table 2).

**Table 2:** Comparison of the characteristics of rMEP-ELISA assay and the commercial ELISA kit

	***Cutoff value***	***Positive***		***Negative***		***PPV (%)***	***NPV (%)***	***Sensitivity (%)***	***Specificity (%)***	***P-value***	***Kappa***

		**TP**	**FN**	**TN**	**FP**						
rMEP ELISA	≥ 0.2^a^	41	2	114	6	87.2	98.2	95.3	95.0	0.046	0.613
Comme rcial Kit	≥ 0.8^b^	43	0	120	0	100	100	100	100		

TP, true positives; TN, true negatives; FP, false positives; FN, false negatives;

PPV, positive predictive value (TP/TP + FP) × 100

NPV, negative predictive value (TN/TN + FN) × 100

a cutoff value is calculated by rMEP ELISA

b cutoff value is calculated by Commercial kit ELISA

## Discussion

The management and treatment of CE with high quality essentially require the early detection of the infection. Immunodiagnostic assessments, especially ELISA, are applied to confirm the imaging techniques’ results (24). Several antigens have been used for ELISA in primary immunodiagnosis, but there are some limitations such as the lack of suitable sensitivity and specificity and difficulties with the standardization (25). Additionally, cross-reactivity with the serum sample from other parasitic infections is another problem (26). So far, there has been no sensitive and specific technique for serodiagnosis of human cystic echinococcosis (27), and recombinant antigens may improve the quality of these tests (28).

Cyst fluid antigens, protoscolex and adult somatic antigens, protoscolex and adult excretory-secretory products, and oncosphere antigens are the widely used antigens in several studies. Nowadays, recombinant proteins, synthetic peptides, or combinations of well-defined antigens are the targets of researchers in this field (29).

B-cell epitopes are the sites recognized by antibodies and are the bases of vaccines and immunodiagnostic tests. Bioinformatics methods can predict the B-cell epitopes based on the properties of 20 amino acids, but the rate of effective prediction is not suitable enough. Hence, to heighten the chance for correct decision, three different kinds of epitope prediction software such as IEDB, Bepipred, and ABCpred were used to predict B-cell epitope.

Ag B (Ag B1, Ag B2, Ag B3, Ag B4, and Ag B5), Ag5, and Ag95 have generally been used in serodiagnosis of human CE, but each of them has revealed some limitations. In the present study, the most immunogenic linear B-cell epitopes in these three epitopes were attached together by “Gly-Ser” linker and used to assess its efficacy in indirect ELISA test using a series of serum samples obtained from CE patients, non-CE patients, and healthy individuals. The results were then compared with an approved commercial kit.

The result of AUC was 0.9964 (95% CI, 0.9914 to 1.001), which indicates our assay has high accuracy for CE diagnosis.

Recombinant proteins and peptides are more reliable for immunodiagnostic and vaccine purposes than natural antigens (30–32). Native AgB was more sensitive than recombinant AgB, with sensitivity of 80 and 68, respectively, but the specificity of recombinant AgB was more than native AgB (33). In a similar study, recombinant AgB had higher sensitivity and specificity than native AgB. The sensitivity and specificity of recombinant AgB were respectively 93% and 99%, and the sensitivity and specificity of native AgB were 60% and 93%, respectively (14). In contrast to these studies, the sensitivity and specificity of native AgB were less than those of recombinant AgB (34).

In Iran, recombinant antigen-ELISA, native Ag B-ELISA, and commercial ELISA kit were compared by another study (2017). The sensitivity and specificity of the recombinant antigen-ELISA, native antigen B-ELISA, and commercial kit were respectively 93% and 92%, 87% and 90%, and 97% and 95% (18). Compared to AgB, recombinant Ag5 and recombinant AgP had a higher rate of sensitivity and specificity than native antigens (35). In the present research, the diagnostic sensitivity and specificity were 95.3% and 95.0%, respectively. Our study showed that rMEP is sensitive and specific for designing immunodiagnostic tests, and it can be used for evaluation of vaccines. AUC for the commercial kit was 1.000, and both its sensitivity and specificity were 100%. The sensitivity and specificity of the commercial kit were more than our rMEP-ELISA, but the differences were not statistically significant (*P*<0.05).

## Conclusion

We assessed a newly established ELISA test with high diagnostic accuracy for human CE diagnosis. However, assessments using a larger number of specific and cross-reactive serum samples must be done to approve its analytical specificity. In our study for the first time, a predicted recombinant protein consisting of *E. granulosus* B-cell epitopes of AgB (8 kDa), Ag5, and Ag95 was expressed and analyzed. Bioinformatics methods can help researchers in finding an immunogenic antigen for the improvement of immunodiagnostic tests. Furthermore, this bioinformatic approach can be used to develop antigens for the diagnosis of other infectious diseases.
